# Jan Stöhlmacher: Damit Vertrauen im Sprechzimmer gelingt: Ein persönlicher Wegweiser für Patienten und ihre Angehörigen

**DOI:** 10.3205/zma001623

**Published:** 2023-06-15

**Authors:** Matthias W. A. Angstwurm

**Affiliations:** 1 LMU University Hospital, LMU Munich, Medizinische Klinik und Poliklinik IV, Munich, Germany

## Bibliographical details

Jan Stöhlmacher

**Damit Vertrauen im Sprechzimmer gelingt: Ein persönlicher Wegweiser für Patienten und ihre Angehörigen**

Publisher: Quintessenz Verlags-GmbH

Year of publication: 2022, 204 pages, price: € 14,90

ISBN: 978-3-86867-602-0

## Review

Trust in the health care system and especially in the doctor who treats them directly leads to an improvement in treatment outcomes [1]. The doctor's behavior influences the patient's trust [2]. In the national average, about 90% of patients have said for years that they are satisfied with their doctor, but 10% of patients have consistently no good or very good relationship with their doctor over years [http://www.kbv.de/html/versichertenbefragung.php]. There are clear variations depending on the origin of patients and medical persons or also between the federal states. Already the privacy at the reception of a practice or an impersonal hospital, for example, plays a major role in whether the patient feels accepted or not. In 2021, 39% of respondents were less satisfied or not at all satisfied with the privacy at the reception. In 2017, 6% of patients said that despite explaining the acute problem or illness, they did not understand it. Unfortunately, patients then ask too few questions so as not to hold up the health service operation or the omniscient staff members.

“It is one of the most important conversations in a person's life when they find out they are seriously ill,” says Jan Stöhlmacher, a hematologist and oncologist. He accompanied two of his closest relatives through these stages of life, observing his own emotions, reflecting on the behavior of himself and, above all, of his caring medical colleagues. Repeatedly, the reaction of the doctors seemed inappropriate to him. These experiences and his individual way of dealing with his own helplessness led to an intensive study of the topic “Trust – what patients and relatives can do for a good climate of discussion”. Thoughts and suggestions for improving communication can be found in the literature, e.g., with oncological patients [3], [4]. But it is precisely the authentic descriptions of situations from the perspective of an affected relative that enable comprehensible emotional reactions promoting empathy for patients, respect their inviolable dignity and point out possible deficiencies in verbal and non-verbal communication. This perspective is certainly new and not yet sufficiently presented in the literature.

For which target groups could the book be of relevant use?

### Patients

The author writes in a way that is very easy to understand even for the layperson, he argues in a concerned and personal way, reveals his individual experiences and assessments, does not shy away from showing emotions and can thus offer assistance for a good sensitive conversation between a patient, a possible accompanying person, and the attending doctors. He avoids drawing up a checklist according to which a conversation is to be prepared or according to which a conversation is to take place. It leaves room for individual situations that meet the needs of patients. In addition to many ideas and recommendations on behavior and strategies, he urges future patients to act as mature, possibly also critically questioning citizens and not as mere recipients of information and instructions for action. A cooperative sensitive conversation, its continuation over several appointments and sufficient time for all questions are the basis of trusting medical activity. Only in this way can the responsible, self-determined patient be involved in decision-making, only in this way is the dignity of the individual patient respected.

### Students

The perspective of patients and their perception of medical activities are not well known to young students without long life experience. As they continue their studies into the practical year, students acquire a great deal of theoretical knowledge and skills, but often do not take on the role of the patient. The unconscious interactions that take place between a doctor and his or her patients can be noticed with many years of experience in the profession of a doctor. In this small book, the reactions of patients and their relatives to verbal and non-verbal communication are shown based on many authentic conversation situations. "In my work as the head of a university outpatient clinic, I have experienced..." (page 14).

This very pragmatic approach to reflecting on conversations and situations allows insights that the author describes very personally. It comes across very well that these interactions depend on the individual personalities of the patients, the possible accompanying persons, and the treating doctors. These situations allow students in advanced semesters and during the practical year to reflect on their own experiences [5]. Thus, any student with increasing competencies in anamnesis techniques and independent, unfortunately often insufficiently supervised, care of patients can use this book to question their approach to managing patients. Naturally, many perspectives of a doctor are presented and reflected coram publicum. This encourages the reader to rethink their own situations and behaviors. This is a very important, perhaps not necessarily intended effect that occurs when reading the book. The reading seems highly recommendable also for PJ students of all disciplines and not only a help for patients as stated in the subtitle.

### Teaching staff and curriculum developer

Finally, the book can also function as an idea generator for generating examination scenarios in which the essential examination objective is not technical knowledge and skills but the competence of communication with the patient. This basic competence of patient-oriented communication with the desirable goal of an equal conversation with the valued patient is specified in the National Competence-Based Learning Objectives Catalogue of Medicine. However, it is possible that these case scenarios are not yet used frequently enough, e.g., in problem-oriented learning, assessed as relevant for the profession of medicine or examined. Since, as described in the book, it is often not major errors at all that impair appreciation and empathic conversation, especially in situations that are significant for the patient, the book can provide suggestions for future examination formats. In practical scenarios, e.g., with drama patients, these can be presented realistically. As an example, in OSCE examinations, students' reactions to patient questions can be tested regarding the appreciation of an individual patient as a competence to be learned. The scenarios described could be the basis for the development of role plays. The book can have a very positive influence on the future of humane, patient-centered individual communication and the ability to make cooperative decisions in medicine.

### General practitioners

In a representative survey conducted by the National Association of Statutory Health Insurance Physicians (Kassenärztlichen Bundesvereinigung) in 2018 on a total of 6,043 randomly selected people, about 16% were not satisfied with their treatment. Of these, only 37% communicated their complaints. 17% did not feel they were taken seriously, 15% felt the doctor and 7% the practice staff were rude [http://www.kbv.de/html/versichertenbefragung.php].

All doctors will reflect on the way they view their relationship with patients by studying the book. The author develops his thoughts from practical situations. These situations are taken from everyday professional life, so that presumably every doctor will have already gone through similar situations. A brief skimming of the book is neither useful nor successful. Since trust does not develop within short moments, but only with repeated longer communications, but is also easily destroyed by careless actions, a quick working through of the book does not seem possible. The book encourages health workers to reflect on their actions and their verbal as well as non-verbal communication. This will probably benefit everyone. To promote self-reflection on conversational situations with patients, to achieve better communication, possibly to a change in previous behavior, the book will be able to provide an impetus over time and hopefully initiate a process.

A trusting communication between doctor and patient also leads to the continuation of the treatment and a change to another colleague can be avoided. The turning away of a patient or the relatives also evokes negative emotions in the attending doctor himself/herself, which can lead to a permanent burden, eventually also in a dissatisfaction or depressive mood. Trusting communication in the consulting room, also on the part of the doctor, is therefore an essential prerequisite for satisfaction with the medical profession, reduces the fear of unpleasant situations or when giving bad news and counteracts burnout [6].

Finally, the book can also be used by a doctor to reflect on a doctor's communication in the role of a friend or relative accompanying the patient's individual person and to prepare the doctor for his new role as an accompanying relative [7].

### Conclusion

Trust is the foundation of every relationship. Trust influences behavior, cooperation, imitation, and further development. In addition, trust in the doctor promotes therapy adherence and thus the long-term outcome of a therapy. However, trust is very easily destroyed, especially in emotionally stressful situations. Self-reflection on one's own behavior with the aim of a trusting, appreciative accompaniment of every person in special situations should be part of every professional development. However, this comes far too short in the daily practice of communication between doctor and patient. The book “Damit Vertrauen im Sprechzimmer gelingt” by Jan Stöhlmacher can be expressly recommended for all those working in the health sector.

## Note

For better readability, the generic masculine is mostly used. The designations of persons always refer to all genders.

## Competing interests

The author declares that he has no competing interests.
